# CD44 is a macrophage receptor for TcdB from *Clostridioides difficile* that *via* its lysine-158 succinylation contributes to inflammation

**DOI:** 10.1080/19490976.2025.2506192

**Published:** 2025-05-18

**Authors:** Zhuo Chen, Wenzi Zhang, Danni Wang, Ruiqin Luo, Yuexin Yao, Xiaoyang Tao, Lu Li, Qin Pan, Xiaoming Sun

**Affiliations:** a School of Basic Medical Sciences, Hubei University of Medicine, Shiyan, China; b Biomedical Research Institute, Hubei University of Medicine, Shiyan, China; c Hubei Key Laboratory of Embryonic Stem Cell Research, Hubei University of Medicine, Shiyan, China; d Hubei Province Key Laboratory of Allergy and Immunology, TaiKang Medical School (School of Basic Medical Sciences), Wuhan University, Wuhan, China

**Keywords:** TcdB, CD44, receptor, succinylation, inflammation

## Abstract

Toxin B (TcdB) is a critical virulence factor in *Clostridioides difficile*-associated disease (CDAD), which activates macrophages to promote inflammation and epithelial damage. However, the mechanism by which TcdB targets inflammation-related receptors on the macrophage surface and the underlying molecular mechanisms remain unknown. The frizzled-binding domain of TcdB (TcdB-FBD) is a promising target of TcdB. Here, FBD was found to trigger macrophage inflammation, similar to TcdB, but did not induce cytotoxicity. Thus, using FBD as a bait protein, macrophage CD44 was identified as an inflammation-related receptor for TcdB/FBD. The role of CD44 was confirmed by CRISPR/Cas9-mediated gene knockout in macrophages and CD44 knockout mice. Using 4-D label-free succinylation quantitative modification proteomics, we demonstrated that TcdB/FBD binds to CD44 in macrophages, promotes CD44 K158 succinylation *via* SUCLG2 suppression, and enhances NF-κB translocation/transcriptional activity, thereby driving inflammation. Finally, blocking the binding of TcdB to CD44 was demonstrated as a favorable strategy for inhibiting TcdB-mediated macrophage inflammation. This study not only provides a new therapeutic target for the prevention and treatment of CDAD but also elucidates a new molecular mechanism underlying the inflammatory effect of TcdB *via* the TcdB/FBD-CD44 axis.

## GRAPHICAL ABSTRACT

**Figure:**
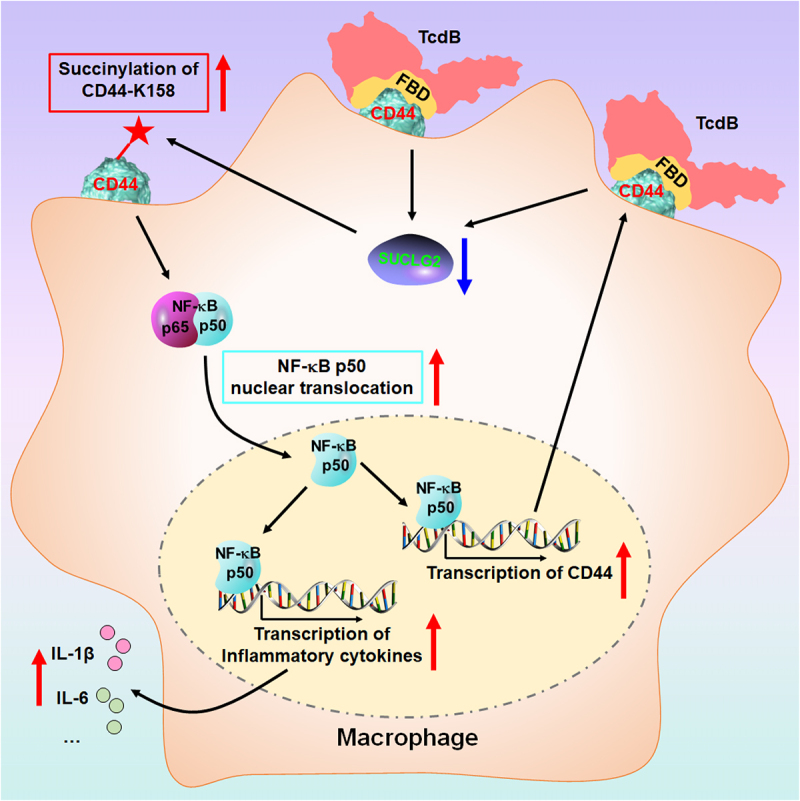

## Introduction

*Clostridioides difficile* is the main cause of antibiotic-associated diarrhea, pseudomembranous colitis, and colonic perforation in developed countries. *C. difficile*-associated disease (CDAD) is associated with more cases involving life-threatening complications.^1–3^ *C. difficile* possesses two exotoxins (TcdA and TcdB) that exert significant virulence. TcdB alone has been shown to induce a full spectrum of illnesses in both humans and animals.^4,5^ Thus, it is often considered the primary toxin responsible for CDAD.^6,7^

**Figure 1.**
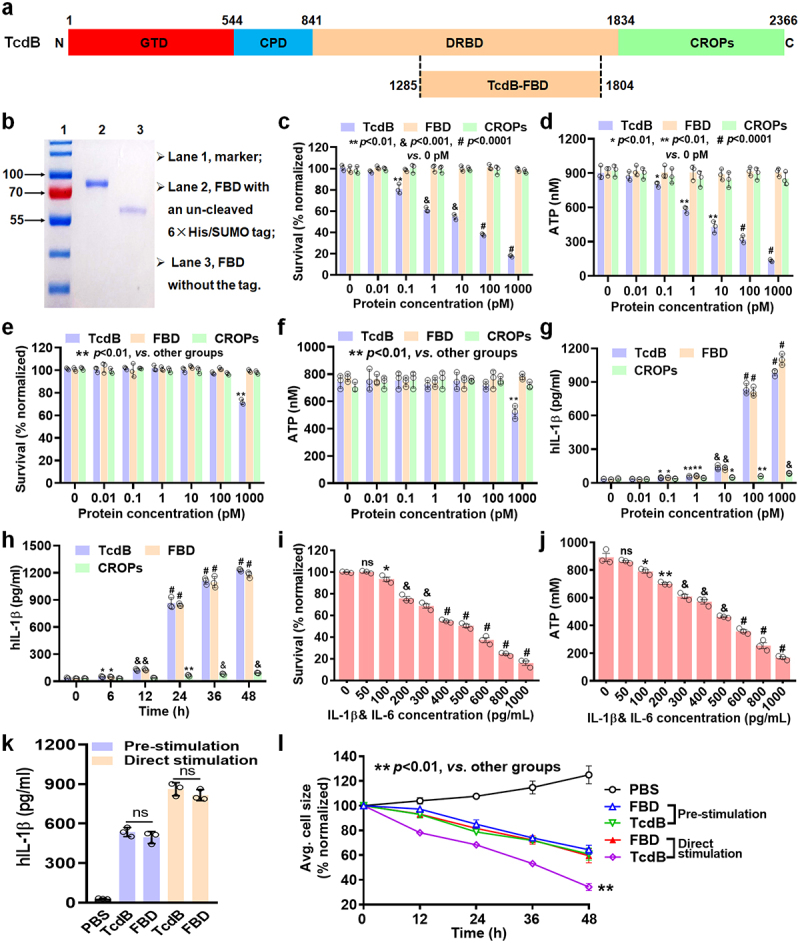
Effect of FBD-mediated macrophage secretion of inflammatory cytokines is comparable to that of TcdB. (a) Schematic diagram showing the domain organization of TcdB. GTD, glucosyltransferase domain (red); CPD, cysteine protease domain (light blue); DRBD, delivery and receptor-binding domain (orange); CROPs, combined repetitive oligopeptides domain (green). (b) The FBD extracted was analyzed by SDS−PAGE. (c–f) cells were treated with a range of TcdB/FBD/CROPs concentrations, and then the survival (c) or ATP levels (d) in caco-2 cells, the survival (e) or ATP levels (f) in THP-1-M*φ*were assessed. (g) THP-1-M*φ* were treated with different proteins for 24 h and then production of IL-1β were measured by an ELISA. (h) THP-1-M*φ* were treated with 100 pM of TcdB, FBD, or CROPs, and the levels of IL-1β was assessed at different time points. (i–j) caco-2 cells were stimulated with different concentrations of inflammatory cytokines (IL-1β and IL-6) for 48 h. The cell (i) viability, and (j) production of ATP by the cells was determined. (k) THP-1-M*φ* were treated with TcdB/FBD (100 pM), after being pre-stimulated or stimulated directly as indicated for 24 h, and the levels of IL-1β were measured. (l) Normalized average cell size over time of caco-2 cells co-cultured with THP-1-M*φ*, which were pre-stimulated or stimulated directly with TcdB/FBD, were analyzed by an HCI system. All data in parts (c–l) are shown as the mean ± SEM (*n* = 3). (ns: *p* > 0.05, *p < 0.05, **p < 0.01, &;*p* < 0.001, #*p* < 0.0001; g: *vs* 0 pM; h: *vs* 0 h; i – j: *vs* 0 pg/mL).

TcdB (~270 kDa) contains four functional domains: the N-terminal glucosyltransferase domain (GTD), the cysteine protease domain (CPD), the central delivery and receptor-binding domain (DRBD), and the C-terminal combined repetitive oligopeptide (CROPs) domain (Figure 1a). It binds to cell surface receptors *via* the DRBD or CROP domains, enters cells, releases GTD, and inactivates small GTPases by glucosylating a key residue, resulting in cytoskeletal dysfunction and eventual cell death.^8,9^ Notably, TcdB exhibits strong cytotoxicity and high affinity for host cells after the deletion of CROPs.^10^ The Frizzled-binding domain (FBD), which mediates the interaction between TcdB and the Frizzled (FZDs) family of colonic epithelial receptors, is located within the DRBD. It is speculated that FBD may be more advantageous for the design of targeted therapeutic agents, and biological functions beyond receptor binding are worth exploring.^11–13^

Macrophages represent a significant proportion of immune cells within the colon tissue.^14,15^ TcdB has also been shown to provoke inflammation in macrophages, thereby exacerbating colonic epithelial tissue damage.^16,17^ Nonetheless, the mechanism by which TcdB targets inflammation-related receptors on the macrophage surface and the underlying molecular mechanism remain unknown. Post-translational protein modifications (PTM) are chemical modifications that occur in proteins after translation and include succinylation, acetylation, crotonylation, and lactylation. PTMs can change the physicochemical properties of proteins that participate in various cellular activities and play an important role in regulating inflammatory processes.^18,19^ Succinylation of a lysine residue can result in a group with a larger structure and induce a significant charge status transformation under physiological conditions, thus significantly changing the structure and function of proteins compared to other PTMs.^20,21^ Therefore, the differential identification of proteomes and post-translational modifications in macrophages with or without TcdB/FBD would unquestionably provide new therapeutic targets against CDAD.

In this study, we found that FBD induced macrophage inflammation comparable to that of TcdB, and identified CD44 on macrophages as an inflammation-related receptor for TcdB/FBD by mass spectrometry. Additionally, using 4-D label-free succinylation quantitative modification proteomics, we demonstrated that TcdB/FBD binds to CD44 in macrophages, promotes CD44 K158 succinylation *via* SUCLG2 suppression, and enhances NF-κB translocation/transcriptional activity, thereby driving inflammation. Finally, we demonstrated that blocking the binding of TcdB to CD44 is a favorable strategy to inhibit TcdB-mediated macrophage inflammation and thus could be developed to prevent and treat CDAD.

## Results

### The non-toxic FBD mediates macrophage inflammatory cytokine secretion with effects comparable to TcdB

TcdB bound to cell surface receptors *via* the DRBD or CROP domains, and FBD was a part of DRBD domain (Figure 1a).^12^ The FBD (Figure 1b) and CROP domains (Figure S1a) were purified and identified using SDS−PAGE. The Caco-2 cell line is commonly employed as an *in vitro* model of the intestinal epithelial barrier for studying TcdB intoxication.^22^ The potent cytotoxicity of TcdB arises from its ability to impede cell proliferation and deplete adenosine triphosphate (ATP).^23^ It also induces the secretion of pro-inflammatory cytokines from macrophages, which exacerbates the breakdown of the epithelial barrier.^14–17^ PMA-differentiated THP-1 cells (THP-1-M*φ*) served as human macrophage models and provided a practical system to investigate TcdB-mediated inflammatory responses in macrophages.^14,15,24^ Cytotoxicity was absent at TcdB concentrations below 0.01 pM in Caco-2 cells (Figure 1c–d), TcdB concentrations below 100 pM in THP-1-M*φ* (Figure 1e–f), and at high concentrations of FBD or CROPs (1000 pM) in either Caco-2 cells or THP-1-M*φ*, but TcdB exceeding 1000 pM altered the cell viability of THP-1-M*φ* (Figure 1e–f). These results indicate that FBD itself is nontoxic to cells, whereas full-length TcdB exhibits potent cytotoxicity.

The production of representative inflammatory cytokines, such as IL-1β or IL-6, by macrophages was induced in a dose- and time-dependent manner by various proteins, including TcdB, FBD, and CROPs (Figure 1g–h; Figure S1b – c). Notably, CROPs or low concentrations of TcdB had a limited impact on cytokine production. However, stimulation with high concentrations of TcdB or FBD (>100 pM) for 24 h resulted in a robust cytokine production. Additionally, significant cytotoxicity was observed only when the inflammatory cytokine (IL-1β and IL-6) concentrations exceeded 100 pg/mL (Figure 1i–j). Thus, we examined inflammatory cytokine production in macrophages treated with 100 pM TcdB/FBD for 24 h in subsequent studies. These results indicate that the newly identified function of FBD is to promote the secretion of pro-inflammatory cytokines from macrophages, with effects comparable to TcdB.

THP-1-M*φ*, in the upper chamber, were co-cultured with Caco-2 cells, which were in the lower chamber, as described in the Methods section, to construct an *in vitro* model of intestinal inflammation.^25^ To simulate the *in vivo* scenario, the TcdB/FBD was added to the culture medium directly, and the damage induced by both the cytotoxicity of TcdB itself and macrophage-generated inflammatory cytokines induced by TcdB, was observed on Caco-2. Notably, FBD itself is nontoxic to Caco-2, but it provoked inflammation of THP-1-M*φ* (Figure 1k; Figure S1d), thereby damage the Caco-2 indirectly (Figure 1l; Figure S1e). When THP-1-M*φ* were pre-stimulated with TcdB/FBD, TcdB did not directly kill Caco-2 due to being washed away, but it did stimulate the secretion of inflammatory cytokines by THP-1-M*φ* (Figure 1k; Figure S1d), thus also indirectly damaging the Caco-2 (Figure 1l; Figure S1e). Taken together, these data indicate that TcdB and FBD can damage intestinal epithelial cells solely by mediating the secretion of pro-inflammatory cytokines from macrophages.

### FBD/TcdB binds to receptor CD44 on macrophages to induce inflammatory cytokine production in vitro

**Figure 2.**
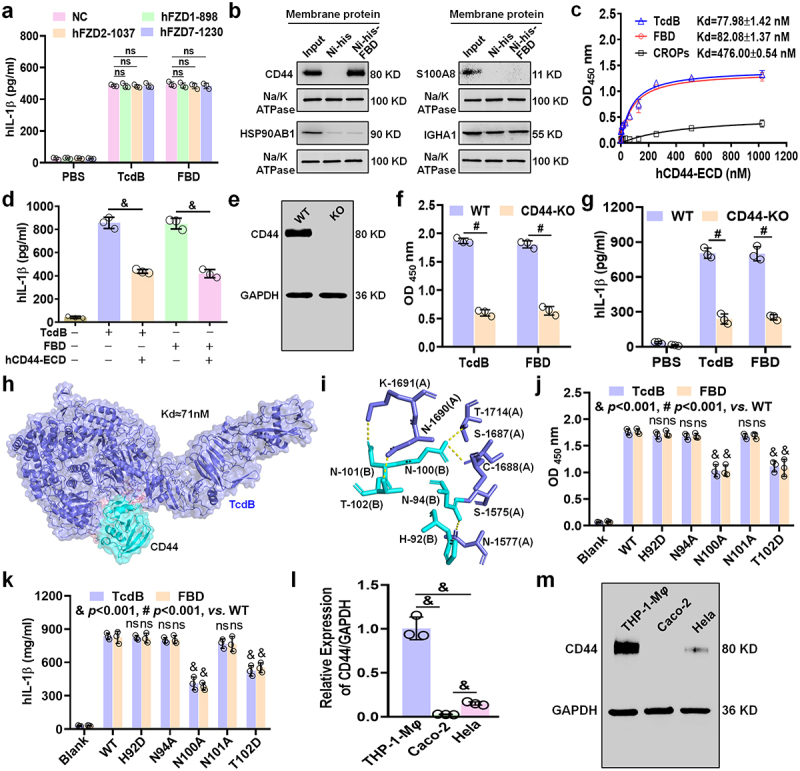
FBD/TcdB binds to CD44 on macrophages to induce inflammatory cytokine production. (a) THP-1-M*φ* were treated with TcdB or FBD (100 pM) for 24 h after knockdown of FZD1/2/7. Levels of IL-1β were measured by an ELISA. (b) An FBD-bead pull-down experiment using membrane extracts of THP-1-M*φ* was assessed by MALDT-TOF-MS, and the identified proteins related to inflammation were analyzed with a WB. (c) ELISA of the binding of human CD44-ECD protein to TcdB/FBD/CROPs. Various concentrations (1–1024 nM) of hCD44-ECD (labeled with fc) were incubated in wells coated with TcdB/FBD/CROPs. (d) THP-1-M*φ* were treated with TcdB/FBD (100 pM) and CD44-ECD (1 μM) for 24 h, and then the levels of IL-1β were measured. (e) Wild-type (WT) and CD44-knockout (CD44-KO) THP-1-M*φ* were identified by WB. (f) Binding of TcdB or FBD to membrane extracts of WT or CD44-KO THP-1-M*φ* as shown by ELISA. (g) WT or CD44-KO THP-1-M*φ* were treated with TcdB or FBD (100 pM) for 24 h, and the levels of IL-1β were measured by an ELISA. Molecular docking predicts the structure and dissociation constant kd value (h) and the interaction sites of TcdB (blue purple) and hCD44 (cyan) complexes (i). (j) Mutations in CD44 that disrupt the interactions of THP-1-M*φ* membrane extracts with TcdB/FBD were demonstrated by ELISA. (k) Wild-type or CD44-mutated THP-1-M*φ* were treated with TcdB or FBD (100 pM) for 24 h, and the levels of IL-1β were measured by an ELISA. (l – m) the expression of CD44 in different cells by (l) qRT-PCR and (m) WB. All data in (a), (c – d), (f – g) and (j – l) are shown as the mean ± SEM (*n* = 3). (ns: *p* > 0.05, &*p <* 0.001, #*p* < 0.0001).

Multiple well-documented receptors of TcdB have been identified, such as FZD1/2/7 receptors of the FZDs family, chondroitin sulfate proteoglycan 4 (CSPG4), and poliovirus receptor-like 3 (PVRL3).^11,26,27^ To investigate the potential dependence of these receptors in mediating inflammation, the corresponding siRNAs that effectively knocked down human (Figure S2a – e) or mouse (Figure S2f – j) FZD1/2/7, CSPG4, and PVRL3 were identified. However, knockdown of these receptors did not result in any significant differences in the production of cytokines by human or mouse macrophages compared to the negative control group (Figure 2a, Figure S2k – q). These results suggest the possibility of other proteins binding to FBD/TcdB in macrophages.

To identify potential membrane receptors of macrophages that bind to FBD/TcdB more accurately, we conducted pull-down assays using FBD-coated magnetic beads but not full-length TcdB-coated magnetic beads, followed by mass spectrometry (MS) analysis to identify differentially bound proteins (Table S1). We used the UniProt website (www.uniprot.org) and published literature to determine the functions of these proteins. From this analysis, we selected four membrane proteins related to inflammation: CD44, HSP90AB1, IGHA1, and S100A8.^28–31^ Co-immunoprecipitation experiments revealed that CD44, a major cell surface receptor, is a novel FBD-associated receptor (Figure 2b). The soluble peptide from the CD44 extracellular domain (CD44-ECD) demonstrated the highest binding affinity to TcdB/FBD, with a Kd values of ~77.98 nmol/L and ~82.08 nmol/L, respectively (Figure 2c), so it inhibited TcdB/FBD-mediated production of inflammatory cytokines in THP-1-M*φ* cells (Figure 2d; Figure S3a). This inhibitory effect was also observed in murine bone marrow-derived macrophages (BMDMs) (Figure S3b−c) because CD44-ECD sequences of mice and humans have high consistency.^32^ Notably, binding assays revealed significantly lower affinity between CD44 and the CROPs domain compared to TcdB or its FBD fragment (which exhibited comparable binding capacities), demonstrating that TcdB-CD44 interaction primarily depends on FBD rather than CROPs (Figure 2c).

To further validate the dependence of the inflammatory status of TcdB/FBD-induced macrophages on CD44, we used the CRISPR-Cas9 technology to generate CD44 knockout (CD44-KO) THP-1 cell lines (Figure 2e). The binding of TcdB/FBD to membrane extracts from CD44-knockout (CD44-KO) THP-1-M*φ* cells was significantly reduced compared to wild-type (WT) THP-1-M*φ* cells (Figure 2f), providing further evidence for the role of CD44 in mediating FBD/TcdB binding. Furthermore, the production of inflammatory cytokines by CD44-KO THP-1-M*φ* cells (Figure 2g; Figure S3d) and by bone marrow-derived macrophages (BMDMs) from CD44-knockout mice (Figures S3e – f) was markedly decreased.

Additionally, TcdB-CD44 interactions (Kd ~71 nmol/L) were predicted by the visualized protein-protein docking conformation (Figure 2h), and we found that multiple groups of residue interactions contributed to the construction (Figure 2i). Point mutations at N100 and T102 in CD44 significantly reduced the TcdB/FBD-binding ability in THP-1-M*φ* cells (Figure 2j), accompanied by a marked decrease in the production of inflammatory cytokines (Figure 2k; Figure S3g). These findings suggest that these residues are critical for receptor recognition and function.

Notably, CD44 is highly expressed in THP-1-M*φ*, significantly lower in HeLa cells, and undetectable in Caco-2 cells (Figure 2l–m). This may be the reason CD44 receptors for TcdB/FBD can be screened in THP-1-M*φ*.

### CD44 functions as an independent receptor for TcdB/FBD in macrophages

**Figure 3.**
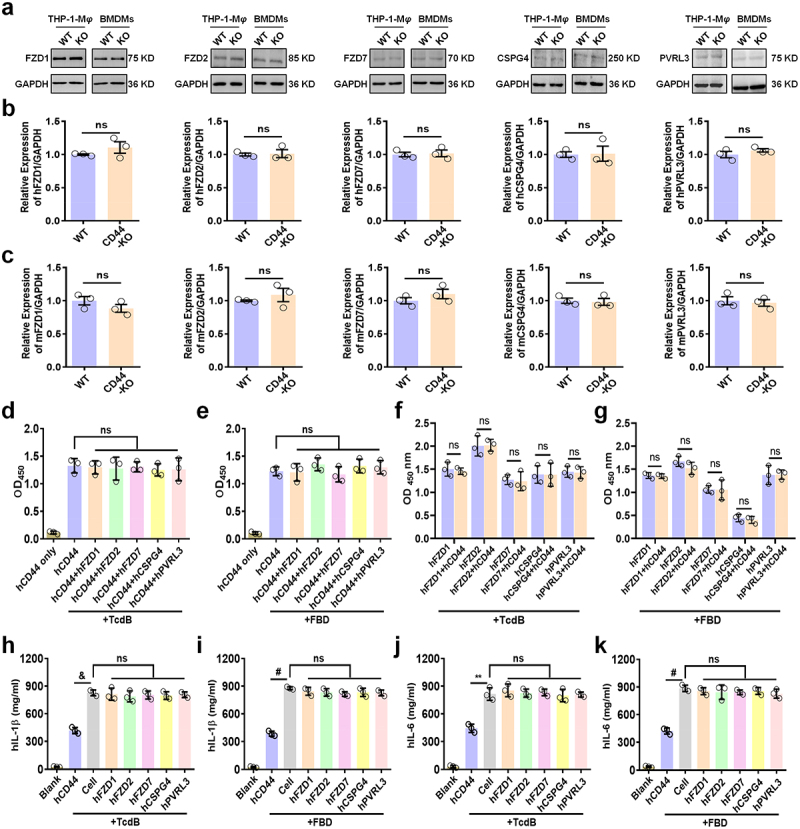
CD44 functions as an independent receptor for TcdB/FBD in macrophages. (a – c) expression of known receptors (FZD1/2/7, CSPG4, PVRL3) in WT versus CD44-KO macrophages analyzed by (a) immunoblotting and (b, c) q-PCR. (d – g) competitive binding assays: (d, e) FZD1/2/7, CSPG4, and PVRL3 did not inhibit CD44 binding of (d) TcdB or (e) FBD, while (f, g) CD44 similarly did not interfere with TcdB/FBD binding to other receptors. (ELISA protocol: wells coated with 1 μM target protein [CD44-ECD or other receptors] were incubated with 1 μM TcdB/FBD, followed by competitor proteins. Binding was quantified by absorbance measurement). (h – k) IL-1β (h, i) and IL-6 (j, k) levels in THP-1-M*φ* treated with TcdB/FBD (100 pM) and receptor proteins (1 μM) for 24 h. All data in parts (b – k) are shown as the mean ± SEM (*n* = 3). ((ns: *p* > 0.05, **p < 0.01, &*p <* 0.001, #*p <* 0.0001).

In CD44-KO macrophages, protein and mRNA levels of known receptors (FZD1/2/7, CSPG4, PVRL3) showed no compensatory changes in both human and murine models (Figure 3a–c), excluding interference from these receptors. Notably, FZD7, CSPG4, and PVRL3 exhibit minimal expression in macrophages (Figure 3a), whereas CD44 is highly expressed on macrophages (Figure 2m). Given that the specific contribution of receptors in a given cell type may depend on their expression levels,^11^ we postulate that these three receptors are unlikely to interfere with CD44 functionality. Additionally, it has been previously confirmed that under stimulation by TcdB/FBD, knockdown of these known receptors does not lead to changes in the levels of cytokines produced by human or mouse macrophages (Figure 2a, Figure S2k – q). However, knockout of CD44 results in a significant reduction in cytokine levels (Figure 2g; Figure S3d – f).

Competitive binding assays demonstrated that TcdB/FBD binding to immobilized CD44-ECD remained unaffected by saturating concentrations (1 μM) of other soluble receptor proteins (Figure 3d–e). Similarly, CD44-ECD did not disrupt TcdB/FBD binding to other receptors (Figure 3f–g), molecularly confirming CD44‘s independent role in mediating TcdB/FBD binding. The low binding affinity between FBD and CSPG4 suggests that CSPG4-TcdB interaction may require additional structural domains. Functionally, TcdB/FBD (100 pM) induced IL-1β/IL-6 secretion in CD44-expressing macrophages, while co-incubation with soluble receptor proteins (including FZD1/2/7, CSPG4, PVRL3) had no effect on cytokine levels, but CD44-ECD suppressed cytokine secretion (Figure 3h–k). These findings collectively demonstrate that CD44 independently mediates TcdB/FBD-induced macrophage activation, unaffected by domain shielding (e.g., CROPs) or co-receptor complexes.

### CD44 is an inflammatory-related receptor for TcdB/FBD in vivo

**Figure 4.**
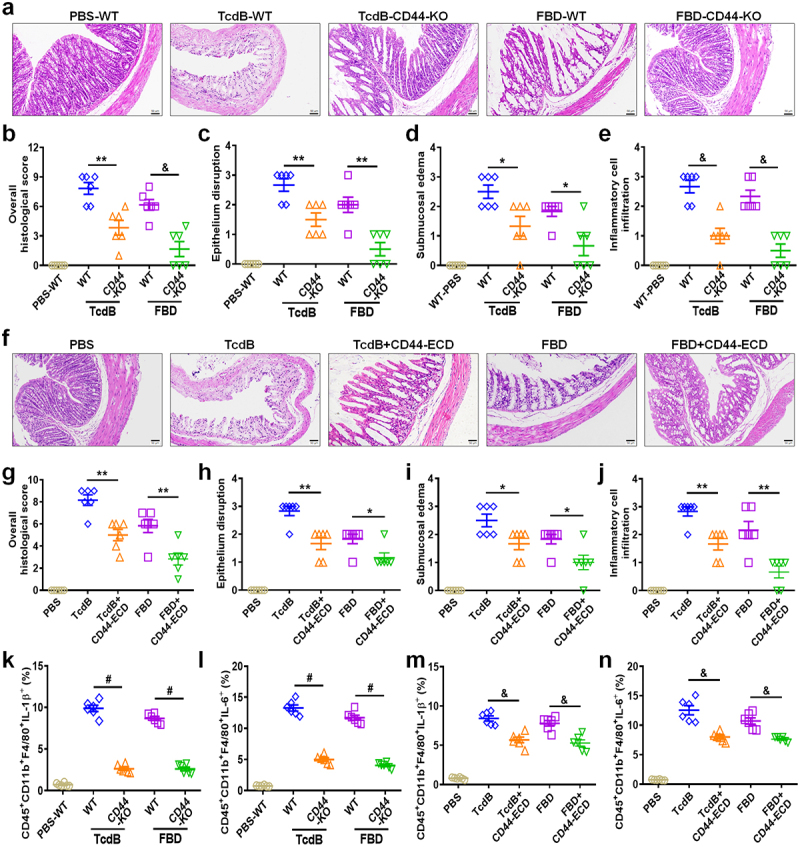
CD44 is an inflammatory-related receptor for TcdB/FBD *in vivo*. (a – j) mouse colonic tissues harvested after intrarectal instillation assays were assessed for pathology through H&E staining. (a) Mice colons were exposed to high concentrations of TcdB/FBD (100 pM) after being stimulated as indicated for 48 h, and representative H&E images are shown. Scale bar represents 50 μm. (b) Overall histology scores are graphed. (c – e) histopathological scores (*n* = 6 mice) for (a) were assessed based on indicated pathological features for epithelium disruption (c), submucosal oedema (d), and inflammatory cell infiltration (e). (f) Mice were exposed to high concentration of TcdB/FBD (100 pM) and supersaturated CD44-ECD (1 μM), after being stimulated as indicated 48 h, and representative H&E images are shown. The scale bar represents 50 μm. (g) Overall histology scores are graphed. (h – j) histopathological scores for (f) were assessed based on indicated pathological features for epithelium disruption (h), submucosal oedema (i), and inflammatory cell infiltration (j). (k – n) FCM statistical analysis of the TcdB- or FBD-induced IL-1β (k, m) or IL-6 (l, n) production by intestinal macrophages in mice. All data in parts (b – e) and (g – n) are shown as the mean ± SEM (*n* = 6). (*p < 0.05, **p < 0.01, &*p* < 0.001, #*p* < 0.0001).

To further investigate the role of CD44 *in vivo*, CD44 KO) mouse models, which showed no intestinal abnormalities, were used. To assess TcdB/FBD-induced colonic damage, we conducted a mouse intrarectal instillation experiment, a widely utilized method for evaluating tissue damage caused by *C. difficile* toxins.^6,33^ Histopathological analysis, as demonstrated by both Hematoxylin-eosin (H&E) staining and histological scoring, revealed that TcdB caused only moderate epithelial disruption, edema, and inflammatory cell infiltration in CD44-KO mice compared to WT mice. Notably, FBD induced minimal tissue damage in CD44 KO mice (Figure 4a–e). In addition, pathological features were mitigated in mice co-administered with TcdB/FBD and CD44-ECD (Figure 4f–j). For the inflammatory cytokines in colonic tissue macrophages induced by TcdB/FBD *in vivo*, the CD44 KO mouse groups exhibited significantly lower levels of IL-1β/IL-6 as compared to WT mice (Figure 4k–l; Figure S4a−c), and the CD44-ECD group inhibited production of inflammatory cytokines (Figure 4m–n; Figure S4d−e). Flow cytometry also revealed that IL-1β^+^ and IL-6^+^ macrophages exhibited elevated CD44 expression following TcdB/FBD challenge (Figure S5a). Quantitative analysis showed a significant increase in CD44 mean fluorescence intensity (MFI) and total macrophage numbers post-stimulation (Figure S5b, c), with the latter change corresponding to inflammatory cell infiltration patterns observed in H&E staining (Figure 4j). CD44-ECD co-treatment reversed both CD44 upregulation and macrophage accumulation (Figure S5b, c).

Collectively, these data establish CD44^+^ macrophages as central contributors to TcdB/FBD-driven intestinal inflammation, where CD44 signaling mediates both pro-inflammatory cytokine production (IL-1β/IL-6) and pathological macrophage expansion, ultimately exacerbating epithelial damage. The therapeutic efficacy of CD44-ECD further validates this mechanistic axis.

### TcdB/FBD induces CD44-dependent inflammasome activation and modulates macrophage phagocytosis

**Figure 5.**
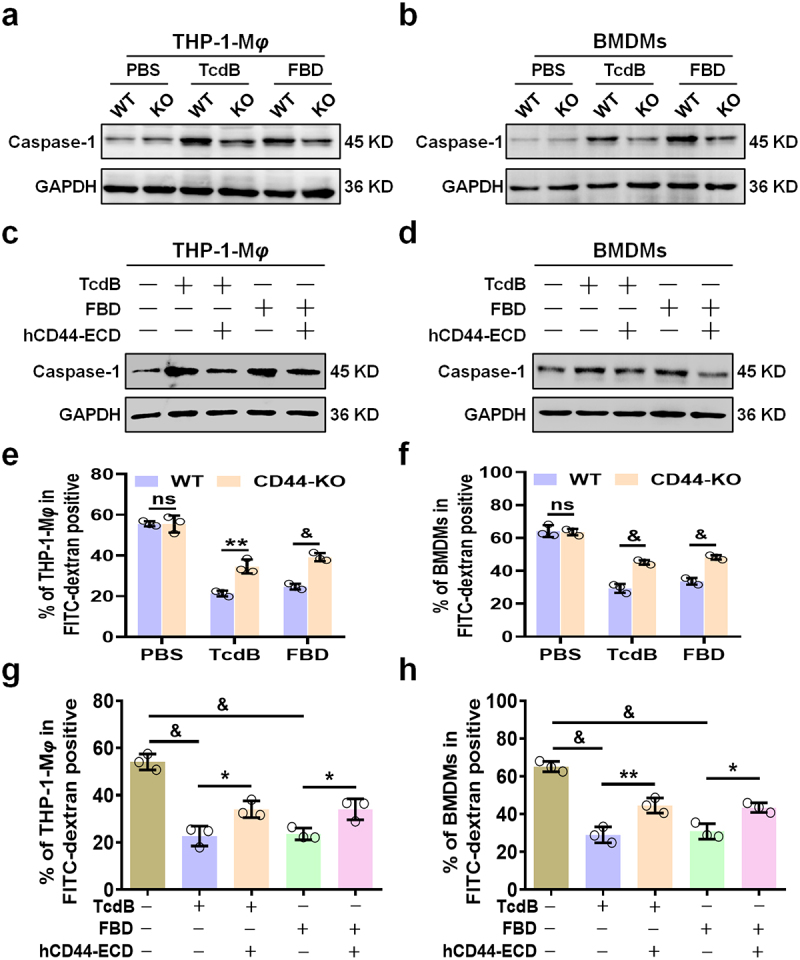
TcdB/FBD induces CD44-dependent inflammasome activation and modulates macrophage phagocytosis. (a – b) Western blot analysis of caspase-1 in (a) THP-1-M*φ* and (b) BMDMs from WT and CD44-knockout macrophages after 24-hour stimulation with 100 pM TcdB or FBD. (c – d) immunoblot showing caspase-1 expression in (c) THP-1-M*φ* and (d) BMDMs treated with 100 pM TcdB/FBD in combination with 1 μM CD44-ECD for 24 hours. (e – f) FITC-dextran uptake assay in (e) THP-1-M*φ* and (f) BMDMs from WT and CD44-knockout macrophages. Cells were treated with 100 pM TcdB or FBD for 24 hours, followed by exposure to FITC-dextran (250 μg/ml) for 1 hour. (g – h) FITC-dextran uptake assay in (g) THP-1-M*φ* and (h) BMDMs co-treated with 100 pM TcdB/FBD and 1 μM CD44-ECD for 24 hours, then incubated with FITC-dextran (250 μg/ml) for 1 hour. Fluorescence intensity was quantified by flow cytometry. Statistical analysis of fluorescence signals. All data are shown as the mean ± SEM (*n* = 3). (ns: *p* > 0.05, *p < 0.05, **p < 0.01, &*p <* 0.001).

To elucidate the molecular mechanisms underlying TcdB/FBD-induced macrophage inflammation, we investigated whether TcdB/FBD could modulate inflammasome activation through its interaction with CD44. Previous studies have reported that TcdB can induce IL-1β release by activating inflammasome.^15^ Consistent with these findings, our results demonstrated that TcdB/FBD significantly activated Caspase-1, a key component of the inflammasome, in both THP-1-M*φ* and BMDMs. However, in CD44-KO macrophages, the expression of Caspase-1 was markedly downregulated (Figure 5a–b). Furthermore, treatment with CD44-ECD also suppressed Caspase-1 activation in WT macrophages (Figure 5c–d). These results suggest that TcdB/FBD promotes inflammasome activation in a CD44-dependent manner, highlighting a novel role for CD44 in mediating TcdB-induced inflammatory responses.

In addition to inflammasome activation, we explored whether TcdB/FBD could modulate macrophage phagocytosis through CD44. TcdB has been reported to inhibit macrophage phagocytosis,^14^ but the role of CD44 in this process remains unclear. Using an FITC-dextran uptake assay, we found that the phagocytic capacity of CD44-KO macrophages was significantly enhanced compared to WT macrophages in the presence of TcdB/FBD (Figure 5e–f; Figure S6a – b). Conversely, treatment with CD44-ECD effectively reversed the TcdB/FBD-mediated suppression of phagocytosis in WT macrophages (Figure 5g–h; Figure S6c – d). These findings indicate that CD44 not only mediates TcdB/FBD-induced inflammation but also plays a critical role in regulating macrophage phagocytosis.

Interestingly, the enhanced phagocytic capacity of CD44-KO macrophages was accompanied by a significant reduction in the production of pro-inflammatory cytokines, such as IL-1β and IL-6 (Figure 2g; Figure S3d – f). This inverse relationship between phagocytosis and cytokine production suggests that CD44 may act as a molecular switch, balancing the inflammatory and phagocytic functions of macrophages in response to TcdB/FBD. When CD44 is absent or blocked, macrophages shift toward a more phagocytic phenotype, potentially as a compensatory mechanism to clear pathogens while minimizing inflammatory damage.

Together, these results demonstrate that TcdB/FBD interacts with CD44 to promote inflammasome activation and modulate macrophage phagocytosis. These findings provide new insights into the dual role of CD44 in mediating both inflammatory and phagocytic responses to TcdB, further underscoring its potential as a therapeutic target for CDAD.

### FBD promotes succinylation of CD44 in macrophages

**Figure 6.**
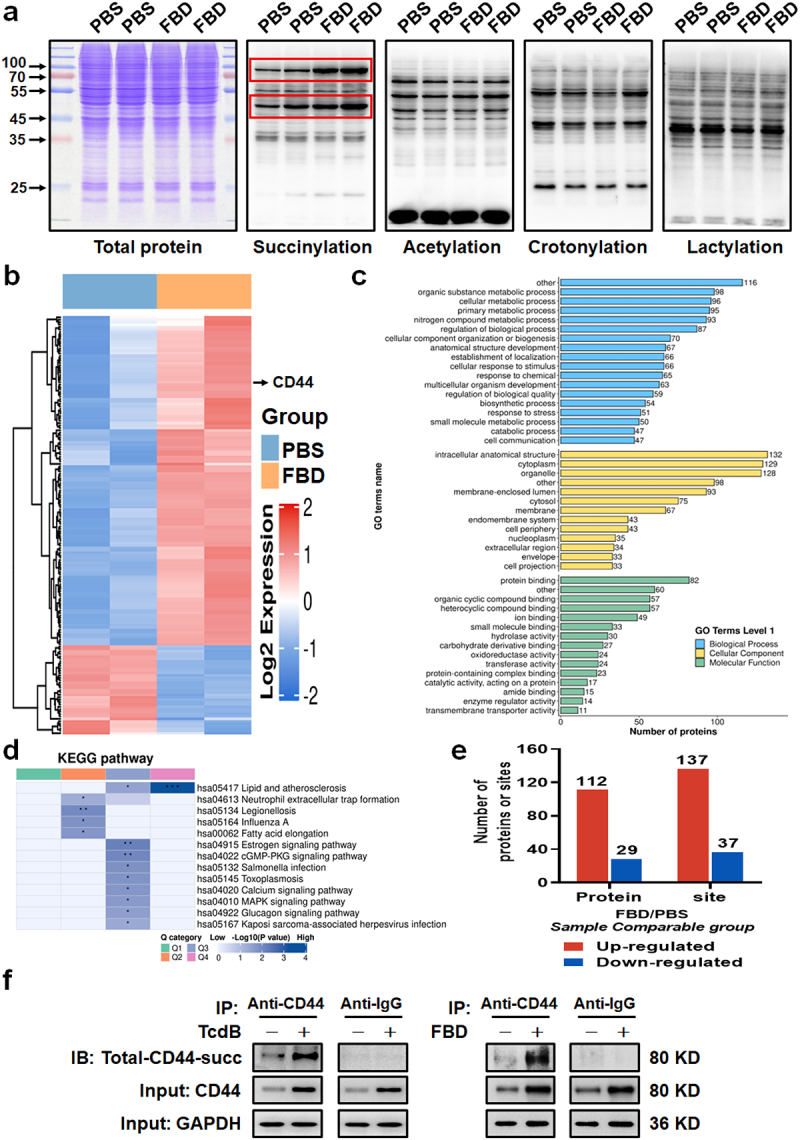
FBD promotes succinylation of CD44 in macrophages. (a) Changes in common PTM levels in THP-1-M*φ* cells caused by FBD were identified. THP-1-M*φ* were treated with FBD (100 pM) for 24 h, and then were detected by WB using specific antibodies against multiple PTMs, including succinylation, acetylation, crotonylation, and lactylation. Red box represents the bands with significant differences, and Coomassie brilliant blue staining was used as a loading control. (b) Heat map for protein succinylation omics screening. THP-1-M*φ* were treated with FBD (100 pM) for 24 h. (c – d) global landscape and functional annotation of FBD-regulated succinylation in THP-1-M*φ*. (c) GO enrichment of quantified differentially modified proteins. Vertical axis represents the secondary functional classification in the primary classification of GO, the horizontal axis represents the number of differentially succinylated modified proteins in the classification, and the different colors represent the primary classification of GO. (d) KEGG pathway enrichment analysis of four groups of samples, Q1 < 0.5, Q2 (0.5–0.667), Q3 (1.5–2.0), and Q4 > 2.0. (e) Summary of differentially quantified sites and proteins. (f) The immunoprecipitation analysis of the total succinylation level of CD44 in THP-1-M*φ* induced by TcdB (left) or FBD (right). Representative western blot lines of total succinyl-CD44 and CD44 proteins in THP-1-M*φ*. The result of the anti-IgG was indicated as a negative control. GAPDH was used as the loading control. Succinylation pan antibodies were used.

Previous reports have indicated that the expression of inflammatory factors in macrophages is closely related to protein PTMs.^18–21^ To further investigate the promotional effect of FBD on inflammatory factors, we performed pan-PTM screening, examining succinylation, acetylation, crotonylation, and lactylation in FBD-stimulated THP-1- M*φ* cells. We found that the succinylation level of total proteins in FBD-treated macrophages increased, whereas other PTMs did not show notable changes (Figure 6a). Subsequently, succinylation proteomics screening was performed with FBD-stimulated THP-1-M*φ* (Figure 6b; Table S2). The identified modified proteins were found in various cell organelles and were involved in several metabolic pathways. GO analysis of upregulated and downregulated proteins, biological processes, and cellular components is shown in Figure 6c. Samples were divided into four groups according to the quantitative ratio of lysine succinylation sites between PBS and FBD-stimulated THP-1-M*φ*: Q1 < 0.5, Q2 (0.5–0.667), Q3 (1.5–2.0), and Q4 > 2.0. KEGG pathway enrichment cluster analysis results of the four groups are shown in Figure 6d. Numerous differentially expressed proteins and lysine succinylation sites were identified in each group, including 137 lysine succinylation sites that were upregulated in 112 proteins, and 37 lysine succinylation sites that were downregulated in 29 proteins (Figure 6e). The results included succinylation of CD44 (Figure 6b; Table S2). Furthermore, we found that the total succinylation levels of CD44 were strongly upregulated in TcdB/FBD-treated macrophages compared to those in the PBS control group (Figure 6f).

### Desuccinylase SUCLG2 mediated CD44 succinylation at Lys158 enhances macrophage inflammatory cytokine production via NF-κB activation

**Figure 7.**
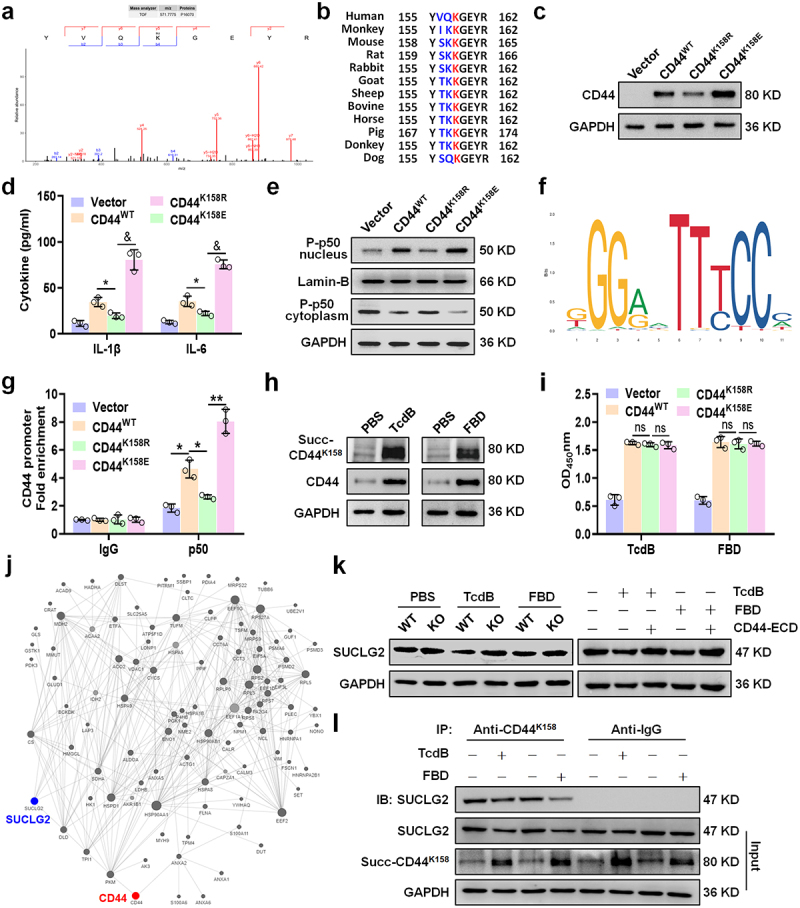
Desuccinylase SUCLG2 mediated CD44 succinylation at Lys158 enhances macrophage inflammatory cytokine production *via* NF-κB activation. (a) Succinylation of CD44 in THP-1-M*φ*, and mass spectrometric verification of CD44 succinylation at K158 (KsuccYVQKGEYR). (b) K158 (marked in red), a CD44 ortholog, is highly conserved in mammals. (c – e) expression of CD44 was measured by WB (c), production of IL-1β and IL-6 was determined by an ELISA (d), and the expression of phosphorylated p50 in the nucleus and cytoplasm was detected by WB (e) in wild-type or CD44-mutated THP-1-M*φ*. (f) Results show the NF-κB-JASPAR binding motif. (g) ChIP assay coupled with qRT-PCR analysis revealed the relative enrichment of NF-κB p50 on the CD44 promoters in THP-1-M*φ*. The Fold enrichment of the ChIP assay was calculated with reference to the control IgG after normalization to the input DNA. (h) Representative western blot lines of succ-CD44Lys158 and CD44 proteins in THP-1-M*φ* after treatment with TcdB/FBD. (i) Mutations in CD44 that did not disrupt the binding of THP-1-M*φ* membrane extracts with TcdB or FBD were demonstrated by ELISA. (j) The protein-protein interaction (PPI) network was constructed using the STRING database based on post-translational modification data. CD44 and SUCLG2 are highlighted in red and blue, respectively. Connecting lines indicate predicted binding, interaction, or complex formation between proteins. (k) Representative western blot bands showing SUCLG2 expression in THP-1-M*φ*. GAPDH served as the loading control. (l) Immunoprecipitation analysis of SUCLG2 binding to succ-CD44Lys158 in TcdB/FBD-treated macrophages. Anti-IgG served as a negative control, with GAPDH as the loading control. All data in (d) and (g – h) are shown as the mean ± SEM (*n* = 3). (ns: *p* > 0.05, *p < 0.05, **p < 0.01, &*p* < 0.001).

Succinylation proteomics screening and mass spectrometry analysis showed that CD44 was succinylated at lysine 158 (K158_succ_) in THP-1-M*φ* (Table S2; Figure 7a). Lys158 is highly conserved among CD44 orthologs in mammals, indicating that it is essential for the function of CD44 (Figure 7b). To elucidate the effect of CD44 K158_succ_ on the secretion of pro-inflammatory cytokines in macrophages, CD44^WT^, CD44^K158R^ (mimicking deletion), and CD44^K158E^ (mimicking succinylation) were stably expressed in THP-1-CD44-KO cells. The routine procedure for succinylation research is to mutate lysine as described above.^19,34^ Notably, compared with the CD44^WT^ and CD44^K158R^ groups, the expression of CD44 was upregulated in the CD44^K158E^ group (Figure 7c) and promoted the secretion of inflammatory cytokines (Figure 7d). The CD44-mediated secretion of pro-inflammatory cytokines is associated with the NF-κB pathway,^35^ and we found that the CD44^K158E^ group had increased nuclear translocation of the NF-κB p50 subunit nuclear translocation (Figure 7e). With JASPAR software, we analyzed the possible binding sites of CD44 promoter and NF-κB (Figure 7f). CHIP-qPCR confirmed the relation between NF-κB p50 and CD44 promoter.^36^ We found that the occupancy of the CD44 promoter was increased by NF-κB p50 in the CD44^K158E^ group. The obtained results showed that the activation of NF-κB p50 can promote the expression of CD44 (Figure 7g). TcdB/FBD robustly increased CD44 succinylation, as validated by succ-CD44Lys158-specific antibodies (Figure 7h). However, neither K158R nor K158E mutations disrupted TcdB/FBD binding (Figure 7i), confirming ligand recognition is succinylation-independent.

To elucidate how TcdB/FBD enhances CD44 succinylation, we have analyzed the protein-protein interaction (PPI) network diagram of CD44 in the succinylation proteomics. Among the predicted upstream succinylase, SUCLG2, a known desuccinylase^37^ could modulate the succinylation of CD44 (Figure 7j). TcdB/FBD suppressed SUCLG2 expression in WT macrophages, while CD44-KO macrophages maintained elevated SUCLG2 levels unaffected by TcdB/FBD. CD44-ECD treatment could reverse the downregulation of SUCLG2 by TcdB/FBD (Figure 7k). Immunoprecipitation results also confirmed that SUCLG2 could interact with CD44succ-Lys158 (Figure 7l), Altogether, suggesting the important role of SUCLG2 in regulating CD44 succinylation.

Our findings reveal that TcdB/FBD binds with CD44 firstly, then downregulates SUCLG2 expression, promoting CD44-K158 succinylation. This enhances NF-κB p50 nuclear translocation, increasing pro-inflammatory cytokine (IL-1β/IL-6) production and further upregulating CD44 transcription. Elevated CD44 expression recruits additional TcdB/FBD ligands, establishing a positive feedback loop that amplifies intestinal inflammation. Crucially, we have demonstrated that the succinylation of CD44 occurs after ligand binding.

## Discussion

TcdB is considered the most critical virulence factor associated with CDAD. It is generally agreed that TcdB leads to cytopathy, apoptosis, and disrupted barrier function in the intestinal tract through Rho GTPase-dependent mechanisms.^2,7–9^ The TcdB-mediated secretion of pro-inflammatory cytokines can also cause tissue damage.^14–17,38^ TcdB causes intestinal tissue damage from the above two aspects, both of which provide nutrition to *C. difficile*, thereby contributing to colonization. Thus, the formation of a positive feedback pathway intensifies the harm caused by *C. difficile*. The literature supports the idea of potent cytotoxic synergy between TcdB and pro-inflammatory cytokines as the driver of CDAD.^39,40^ However, the FBD of TcdB does not have glucosyl transferase activity,^8,12,13^ and without inducing cytotoxicity, it exhibits additional functions beyond receptor binding. Specifically, it elicited the release of pro-inflammatory cytokines in macrophages (Figure 1c–h; Figure S1b – c). This may represent an additional mechanism by which the FBD regions or TcdB participate in the pathophysiology of CDAD.

Recent research has uncovered how TcdB induces pro-inflammatory cytokines by intracellular mechanisms.^14,15,24^ Nevertheless, the upstream machinery responsible remains unclear, and it is possible that known membrane receptors (FZD1/2/7, CSPG4, PVRL3) may not be involved in the secretion of pro-inflammatory cytokines (Figure 2a; Figure S2k−q). We identified CD44 on macrophages as an inflammation-related receptor of TcdB based on the following evidence: (1) CD44-ECD has a high binding ability to TcdB/FBD (Figure 2b, c), thereby inhibiting macrophage-derived inflammation induced by TcdB/FBD *in vitro* (Figure 2d; Figure S3a – c) and *in vivo* (Figure 4m–n; Figure S4d – e). (2) The binding of TcdB/FBD to membrane extracts of CD44-knockout macrophages was significantly reduced (Figure 2f), and the production of inflammatory cytokines was also significantly reduced *in vitro* (Figure 2g; Figure S3d−f) and *in vivo* (Figure 4k–l; Figure S4b – c). (3) Point mutations in several important sites of CD44 on THP-1-M*φ* reduced the TcdB/FBD-binding ability against THP-1-M*φ* (Figure 2j), and the production of inflammatory cytokines was decreased (Figure 2k; Figure S3g). (4) The expression of known receptors remained unchanged in both WT and CD44-KO macrophages (Figure 3a–c). These soluble receptors neither inhibited TcdB/FBD binding to CD44-ECD (Figure 3d–e) nor suppressed TcdB/FBD-induced IL-1β/IL-6 secretion in macrophages (Figure 3h–k).

Identification of the TcdB receptor is an important step toward understanding the underlying molecular mechanisms involved in the interplay between toxins and host cells.^26,41^ Thus, the identification of five receptors for TcdB has been reported using full-length TcdB in HeLa or Caco-2 cells.^11,26,27,33,42^ However, we presumed that using a truncated domain of TcdB, rather than full-length TcdB, would more precisely identify relevant receptors. Moreover, the specific contribution of the receptors in a given cell type may be influenced by their expression levels.^11^ Thus, the aforementioned method may result in the omission of a crucial receptor that interacts with TcdB, such as CD44, which is significantly increased in macrophage as compared to HeLa or Caco-2 cells (Figure 2l–m). In addition, the above report used a genome-wide CRISPR/Cas9 screen for receptors screen,^11,26,33,42^ which we noticed is highly advanced. However, this method is dependent on the stressful environment of toxin-induced cell toxicity, as it may not work for proteins with low toxicity such as FBD. Thus, screening for proteins that interact with FBD using MS may be more appropriate. CD44 may be the first example of a receptor for TcdB screened in immune cells. Given the propensity of CDAD to recur after recovery,^43^ we postulated that a novel TcdB/*C. difficile*-related receptors in immune cells will aid in the development of novel therapeutics for CDAD.

CD44, a type I transmembrane glycoprotein, exhibits upregulation in immune cells during the secretion of pro-inflammatory cytokines, as evidenced by its high expression on colonic macrophages in mice with colitis.^28,32,35^ As a prominent cell surface receptor, CD44 plays a pivotal role in regulating inflammatory signaling *via* its extracellular domain to bind ligands.^32^ Notably, CD44 demonstrates the ability to bind to diverse pathogenic factors, such as a lipoglycan present on the surface of mycobacteria, thereby facilitating the release of inflammatory factors from macrophages.^44^ The receptors offer a cellular “doorknob” for TcdB to interplay with cells, which may present potential new molecular targets for the treatment of CDAD.^41^

PTMs are crucial for protein structure and function. In recent years, it has been confirmed that succinylation significantly alters the activity and structure of proteins and participates in the regulation of macrophage-mediated inflammatory responses.^18–21^ To elucidate the related mechanisms that regulate inflammation following the binding of TcdB/FBD-CD44, we found that lysine at position 158 on CD44 was highly succinylated in macrophages induced by TcdB/FBD. Increased succinylation at lysine 158 upregulates the expression of CD44 and promotes the secretion of pro-inflammatory cytokines. In contrast, mimicking the desuccinylation of CD44 K158 inhibited the inflammatory (Figure 7a–h). Our research sheds new light on the molecular basis of macrophage-mediated inflammation induced by TcdB/FBD, and targeting succinylated CD44 may provide a novel strategy for regulating inflammation in other diseases.

While CD44 has been implicated in lipid raft partitioning and co-receptor functions for certain pathogens (including as a putative co-receptor for *C. difficile* CDT toxin),^45,46^ our study establishes that TcdB/FBD triggers macrophage inflammatory signaling through a fundamentally different pathway. Three key observations support this conclusion: (1) CD44 shows no detectable redistribution to lipid rafts following TcdB/FBD stimulation (Figure S7a); (2) Neither point mutations (N100A/T102D) nor succinylation-mimetic variants (K158E) of CD44 alter its lipid raft partitioning (Figure S7b – d); and (3) The TcdB/FBD-CD44 axis activates inflammation through a clearly defined SUCLG2-succinylation-NF-κB cascade (Figure 7). These findings collectively demonstrate that CD44 functions as a bona fide primary receptor for TcdB/FBD in macrophages, with its signaling mechanism being distinct from both its proposed co-receptor roles and its established lipid raft-associated functions in other biological contexts.

Among the proteins screened and analyzed by MS, other proteins aside from those studied here may also be of concern (Table S1), as well as other proteins in succinylation omics screening (Table S2). The residue-binding sites of TcdB-CD44 displayed through the protein-protein docking conformation may not be comprehensive, and it will be of great significance to reveal the structure of the TcdB-CD44 receptor-binding interface through cryo electron microscopy. This study focused only on the direct effects of TcdB on host tissues. The potential therapeutic effects of CD44-ECD on CDAD will be assessed in *C. difficile* at a later stage. This is a limitation of the present study. While the documented differences between THP-1 cells and primary human monocytes,^47^ our experimental data demonstrate that THP-1-M*φ* and BMDMs exhibit highly consistent responses to TcdB/FBD-induced cytokine secretion (Figure 2d, g; Figure S3a – f). This alignment with established models in the field supports the utility of THP-1-M*φ* as a practical system for studying TcdB-mediated macrophage inflammatory responses.^14,15,24^

Exaggerated inflammation is associated with worse outcomes in patients with CDAD, and *C. difficile* may thrive in inflamed environments. Therefore, a promising therapeutic strategy against CDAD involves host-oriented therapy aimed at regulating the immune microenvironment.^14–16,48,49^ This study demonstrates that CD44 is an inflammation-related receptor for TcdB. TcdB binds to CD44 in macrophages, promotes CD44 K158 succinylation *via* SUCLG2 suppression, and enhances NF-κB translocation/transcriptional activity, thereby upregulates the expression of CD44 and promotes the secretion of pro-inflammatory cytokines. Therapeutically, small-molecule tools can be used to block TcdB-CD44 binding, leading to a reduction in the production of inflammatory cytokines, resulting in decreased damage to intestinal epithelial cells by TcdB *in vitro* and *in vivo*. Our study identified a novel therapeutic target for the treatment of CDAD.

## Materials and methods

### Cloning, expression, and purification of recombinant protein

The nucleic acid sequences encoding TcdB-FBD (residues 1285–1804) and TcdB-CROPs (residues 1834–2366) from the genome of ATCC strain 43,255 were cloned into a modified pET28a vector featuring a 6×His-SUMO tag at the N-terminus. Recombinant proteins were expressed and purified in the *Escherichia coli* strain BL21 (DE3) (Invitrogen, Carlsbad, CA, USA) following previously established protocols.^8,12^ The additional His-tag at the N-terminus of TcdB-FBD facilitated the pull-down and cell surface-binding assays. After cleavage of the His-SUMO tag by SUMO proteases, the unlabeled TcdB-FBD was further purified for use.

### Cell culture and macrophage transfection

THP-1 human monocytic, Caco-2 human colorectal adenocarcinoma, and HeLa human cervical cancer cell lines were procured from the China Center for Type Culture Collection (CCTCC, Wuhan, China). They were confirmed to be free of *Mycoplasma* contamination and were authenticated by STR profiling (Haixing Bioscience, China). All cell lines were maintained in their respective medium under humidified conditions in a CO_2_ incubator at 37°C.

THP-1 cell suspensions were cultured in RPMI-1640 medium supplemented with 10% fetal bovine serum (Cat# 16000–044, FBS, Gibco) and seeded into experimental vessels (6-, 12-, or 24-well plates) at a density of 1 × 10^6^ cells/mL. Following seeding, cells were stimulated with 100 ng/mL phorbol 12-myristate 13-acetate (PMA, Cat# P1585, Sigma-Aldrich, *St*. Louis, MO, USA) for 24 h, after which they were washed and cultured in fresh medium for an additional 24 h. PMA stimulation serves to transform THP-1 cells to a macrophage-like phenotype.^50^

Murine bone marrow-derived macrophages (BMDMs) were used as *in vitro* mouse macrophage model. Bone marrow cells were procured from the femurs and tibias of C57BL/6 mice and cultured for 7 days in DMEM/F12 medium (Gibco) supplemented with 10% FBS (Cat# mu001SR, QmSuero/Tsingmu Biotechnology, Wuhan, China), 50 ng/mL M-CSF, 100 U/mL penicillin, and 100 µg/mL streptomycin to attain the resting state of macrophages.^44,50^ M-CSF was purchased from Chamot Biotechnology Co., Ltd (Cat# CM061).

Transient transfection of siRNA was performed using the Advanced DNA/RNA Transfection Reagent (Cat# AD600100, ZETA^TM^ LIFE, San Francisco, CA, USA) according to the manufacturer’s instructions. The corresponding siRNA sequences for the knockdown of human or mouse FZD1/2/7, CSPG4 and PVRL3 in Table S3.

### Mice

C57BL/6 mice (6–8 weeks, female, specific pathogen-free) were purchased from the Laboratory Animal Resources Center at Hubei University of Medicine (Shiyan, China). The CD44 KO mice (Originating from C57BL/6 mice) were generated at the Model Animal Laboratory Center of Wuhan University (Wuhan, China) and bred at the Hubei University of Medicine. The mice were housed under specific pathogen-free conditions with free access to drinking water and food during the experiments.

### Cell viability and cell damage analysis

Cell viability was assayed using a CCK-8 kit, according to the manufacturer’s instructions (Cat# C00428, Beyotime, China). The ATP assay kit was purchased from Beyotime (Cat# S0027), and the assay was performed according to the manufacturer’s instructions. The cells were lysed, harvested, and centrifuged to remove cell debris. The supernatant was then added to the substrate solution and luminescence was recorded using an illuminometer (SpectraMax i3×, Molecular Devices, Silicon Valley, CA, USA). Human IL-1β and IL-6 were purchased from SinoBiological (Cat# 10139-H07E; Cat# 10395-HNAE).

To simulate the effects of macrophages located within the intestinal epithelium on intestinal epithelial cells *via* soluble cytokines, an *in vitro* model of intestinal inflammation was constructed using a coculture system. Human intestinal epithelial-like Caco-2 monolayers and activated macrophage-like THP-1 cells were utilized in this study as described previously.^25^ Specifically, THP-1 cells were transformed into a macrophage-like phenotype by treatment with PMA following the above method. Caco-2 cells were seeded in 24-well plates (Corning Costar) and cultured to ~ 90% confluence, and co-cultured with THP-1-M*φ* in a Transwell system (8-μm, 24-well format, Corning). The ratio of Caco-2 cells to THP-1-M*φ* was 2:1. In certain situations, to observe the direct damage caused by macrophage-generated inflammatory cytokines to Caco-2 cells, THP-1-M*φ* pre-stimulated with TcdB or FBD were used. Specifically, THP-1-M*φ* were exposed to TcdB or FBD for 6 h in humidified incubator. The cells were washed three times with PBS, harvested, counted, placed in the upper chamber of the Transwell system, and co-cultured with Caco-2 cells in the lower chamber. In certain situations, we observed the dual damage caused by TcdB and macrophage-generated inflammatory cytokines in Caco-2 cells to simulate a more realistic *in vivo* scenario. Specifically, THP-1-M*φ* were harvested with a cell scraper, counted, and placed in the Transwell system upper chamber. TcdB or FBD were added directly to the culture medium and co-cultured with Caco-2 cells in the lower chamber. The cells were cultured in a high-connotation cell imaging analysis system (HCI system, Operetta CLS™, PerkinElmer, Waltham, MA, USA), and their exposure to humidified conditions in a CO_2_ incubator at 37°C for 48 h was confirmed. Real-time cellular microscopy images were captured every 6 h and the area of Caco-2 cells in each group was automatically calculated. The calculated cell area values from the six sites were averaged, and the averages from different wells were considered as technical replicates. The cell area was then normalized to the initial average area, and plotting and statistical analyses (one-way analysis of variance [ANOVA]) were performed using GraphPad Prism 8.0. The results were plotted to illustrate representative examples from three independent experiments.

### Quantitative reverse transcription PCR (qRT-PCR)

Total RNA was extracted from cells using TRIzol Reagent (Cat# 15596026, Life Technologies, Carlsbad, CA, USA). According to the manufacturer’s instructions, the ReverTra Ace-α- First Strand cDNA Synthesis Kit (Cat# RR036A–1, Takara, Japan) was used to synthesize first-strand cDNA from the mRNA in the total RNA sample, and the assays were performed for quantification of gene expressions of human or mouse FZD1/2/7, CSPG4, PVRL3 and CD44 using SYBR Green real-time PCR Master Mix kit (Takara, Japan). qRT-PCR was performed using a CFX96 Touch Real-Time PCR System (CFX 96, Bio-Rad, Hercules, CA, USA). Target gene expression was normalized to glyceraldehyde 3-phosphate dehydrogenase (GAPDH). All primers used for the target and reference genes are listed in Table S4. The average threshold cycle (CT) values from triplicate PCR were normalized to the average CT values for GAPDH from the same cDNA sample.

### FBD-bead pull-down assay

THP-1-M*φ* (4 × 10^7^/mL) were cultured, and then the cell membrane proteins were extracted. Ni-beads (20 μg) were conjugated with 20 μg FBD-His or His protein in 500 µL PBS, and washed three times with PBS. The FBD-conjugated beads were incubated with 2 mg cell membrane proteins in 500 µL binding buffer at 4°C for 12–16 h by gentle shaking, as previously described. Unbound proteins were removed from the beads by washing five times with the washing buffer.^50^ Proteins adsorbed on the washed beads were identified using matrix-assisted laser desorption ionization time-of-flight mass spectrometry (MALDT-TOF-MS, TripleTOF^5600+^, SCIEX, Framingham, CA, USA).

### Immunoprecipitation and western blotting

To detect the interaction of FBD with CD44, IGHA1, HSP90AB1, and S100A8, THP-1-M*φ* were treated with 0.5 μM FBD with a His-tag for 6 h, and then washed three times with PBS to remove nonspecific proteins. Cell membrane proteins were extracted and subjected to immunoprecipitation using antibodies against His. The proteins were then analyzed by immunoblotting.^50^ The primary antibodies were anti CD44 (Cat# 37259, Cell Signaling Technology, Danvers, MA, USA); anti HSP90 alpha/beta (Cat# MA5–33168, Thermo Fisher, Waltham, MA, USA); anti IGHA1 (Cat# MA5–31774, Thermo Fisher); anti S100A8 (Cat# MA5–36007, Thermo Fisher) antibodies. The reference antibody used was the Na, K-ATPase antibody (Cat# 23565, Cell Signaling Technology). HRP-conjugated secondary antibodies were purchased from Affinity Biosciences Inc (Cincinnati, OH, USA). Chemiluminescence was detected using ECL Plus western blotting reagents.

To analyze the total succinylation levels of CD44 in macrophages induced by TcdB/FBD, THP-1-M*φ* were treated as indicated for 24 h. Whole cells were harvested and suspended in an ice-cold lysis buffer. Lysates were sonicated on ice and incubated for 30 min before being centrifuged at 12,000 rpm for 30 min at 4°C. A protein A/G immunoprecipitation kit (Cat# IK-1004, Biolinkedin, China) was used according to the manufacturer’s instructions. The cells were then analyzed by immunoblotting.

Nuclear and cytoplasmic proteins were extracted separately using the Nuclear and Cytoplasmic Protein Extraction Kit (P0027, Beyotime). The anti-phospho-NF-κB1 p105/p50 (Cat# 13586, Cell Signaling Technology) antibody was used in immunoblotting. GAPDH or Lamin-B (Cat# P20700, ZEN BIO) was used as an internal reference control to quantify the relative protein expression.

The other Primary antibodies used in this study were as follows: (Cat# 22915–1-AP), CSPG4 (Cat# 55027–1-AP), PVRL3 (Cat# 11213–1-AP), SUCLG2 (Cat# 14240–1-AP), FZD2 (Cat# 24272–1-AP), and FZD7 (Cat# 16974–1-AP) from Proteintech Group (Wuhan, China); FZD1 (Cat# PA5–86484) from Thermo Fisher Scientific; Flotillin-1 (Cat# ab41927) from Abcam (Cambridge, UK).

### Lipid raft isolation

Lipid rafts were isolated from scraped cells using the ReadyPrep™ Protein Extraction Kit (Signal) (Cat#1632087, Bio-Rad, Hercules, CA, USA). Briefly, cells were washed, processed with S1/S2 buffers (200 μL each), and centrifuged (12,000 rpm, 20 min, 4°C). Total protein (50 μL) was collected; supernatant and pellet were separated into non-lipid raft and lipid raft fractions. The lipid raft pellet was sonicated in IP lysis buffer. Protein fractions were analyzed by Western blot for CD44, with Flotillin-1 as a lipid-raft marker (positive control).

### Generating CD44 KO cell lines

The CD44 gene was ablated in THP-1 cells using CRISPR-Cas9 technology, as previously described.^51^ To specifically target exon 2 of the CD44 gene, two guide RNA sequences, CTTTGTTGCAGAGCAATCAATGG and CTTGGCCAAATTAATACGTGGGG, were selected *via* the CRISPR design tool available at http://crispr.mit.edu. The presence of insertions or deletions in CD44-targeted clones was assessed by PCR amplification with primers flanking the exon (forward: 5'-GGGGGTTAGACAAGGGTGTT-3'; reverse: 5'-CTTGCTCTTCTTGTGCACCG-3').

### Generation of stable cell lines

Stable cell lines were established using a lentivirus system, as previously described.^34^ In brief, full-length wildtype (WT) cDNA with a Flag tag or cDNA containing point mutations of the CD44 gene (K158E/K158R, H92D, N94A, N100A, N101A, and T102D) was synthesized by GenePharma (Shanghai, China) and cloned into the specified vectors LV5 (EF-1a/GFP&Puro) (GenePharma). These constructs were then transfected into THP-1-CD44-KO cells using the ZETA^TM^ LIFE Transfection Reagent. To select stable transfected cell lines, 1 µg/mL puromycin (Solarbio, Beijing, China) was added for a duration of two weeks.

### Enzyme-linked immunosorbent assay (ELISA)

THP-1-M*φ* or BMDMs (1 × 10^6^/mL) were cultured with different proteins, such as TcdB, FBD, or CROPs, for 24 h. The cells were incubated with 100 pM of these proteins and the culture supernatants were collected at different time points. For pre-stimulation, cells were exposed to TcdB or FBD for 6 h in a humidified incubator. The cells were then washed thrice with PBS and cultured for 24 h. The cell culture supernatants were collected, and cytokine levels were determined using ELISA kits purchased from 4ABiotech (China), including human IL-1β (Cat# CHE0001), human IL-6 (Cat# CHE0009), mouse IL-1β (Cat# CME0015), and mouse IL-6 (Cat# CME0006), according to the manufacturer’s protocols.

To evaluate the binding of FBD/TcdB to cells, cell membrane proteins were extracted (Cat# P0033, Beyotime) and coated onto an ELISA plate. After blocking with 200 μL of 0.1% BSA at 37°C for 1 h, FBD-related groups were incubated with an anti-His-tag antibody, and the TcdB-related groups were incubated an anti-TcdB-GTD antibody (Cat# ab83066, Abcam). After washing, the HRP-labeled secondary antibodies were added to the respective groups and incubated for 30 min at 37°C. After adding the substrate and stop buffer, the absorbance was measured at 450 nm using a microplate reader.

To further investigate the competitive binding interactions between FZD1/2/7, CSPG4, PVRL3 or CD44 with TcdB/FBD, FZD1/2/7 and CSPG4 (Cat# 5988-FZ; Cat# 1307-FZ; Cat# 6178-FZ; 2585-PG. R&D Systems, Minneapolis, MN, USA) or the extracellular domain of CD44 (CD44-ECD, amino acids 21–220) and PVRL3 (Cat# 12211-H02H; Cat: 10852-H02H; SinoBiological, China) were used in an ELISA. Native TcdB was obtained from Abcam (Cat# ab124001). Various receptor proteins were separately immobilized onto ELISA plates, followed by sequential addition and incubation of TcdB/FBD, as previously described.^44^

### Proteomic quantification of lysine succinylation

THP-1-M*φ* were exposed to FBD at a concentration of 100 pM or to PBS for a duration of 24 h. To perform pan-PTM screening, we used succinylated pan-antibody (Cat# PTM-401), acetylated pan-antibody (Cat# PTM-102), crotonylated pan-antibody (Cat# PTM-502), and lactylated pan-antibody (Cat# PTM-1401RM) to examine multiple post-translational modifications. All antibodies were obtained from PTM Biolabs (Hangzhou, China). Subsequent affinity enrichment and mass spectrometry-based quantitative proteomics were used to quantify the dynamic alterations in the lysine succinylome, which was conducted by PTM Biolabs. Comprehensive bioinformatic analyses were subsequently performed to annotate quantifiable targets, including protein annotation, functional classification, functional enrichment, and cluster analysis.^34^ We also obtained the succ-CD44Lys158 customized antibody (Cat# CO0308, PTM Biolabs).

### Protein-protein (TcdB-CD44) docking simulation

The crystal structures of TcdB (6C0B) and CD44 (1UUH) were retrieved from the Protein Data Bank (https://www.rcsb.org/) and subjected to HDOCK. All parameters were selected by default. For a comprehensive analysis, we selected complex.1 and applied LigPlot^+2.2.4^ to identify functional residues, including hydrogen bonding interactions, salt bridges or hydrophobic interactions, and PyMol^2.2.0^ to visualize the protein-protein docking conformation. In LigPlot^+2.2.4^, TcdB is labeled A and CD44 is labeled B. In PyMol, TcdB1 is represented as blue-purple cartoons, CD44 is shown as cyan cartoons, and their binding sites are displayed as a series of pink sticks. The Kd of combined proteins were calculated by Prodigy (https://bianca.science.uu.nl/prodigy/).

### Chromatin-immunoprecipitation-polymerase chain reaction (ChIP-PCR)

THP-1-M*φ* from the stable cell lines of the CD44 gene (WT, K158E, K158R) were prepared for the chromatin immunoprecipitation assay by using a ChIP Assay Kit (Millipore, MA, USA) according to the manufacturer’s protocol. The fragmented chromatin samples were immunoprecipitated with antibodies specific for NF-κB p50 or control IgG (Millipore), reverse cross-linked, purified, and analyzed using qRT-PCR.^36^ The following primer sets were used for the amplicons of the human CD44 promoter regions: 5’-TGTGTAACTCACCAGGCAAG-3’ (forward) and 5’-CATCCACCCATACGTTCATC-3’ (reverse).

### Mouse intrarectal instillation assay

To evaluate colonic damage induced by TcdB/FBD, we conducted an intrarectal instillation experiment in mice, a method extensively used to assess tissue damage caused by *C. difficile* toxins as documented in previous studies.^6,33^ Mice were randomized prior to the experiment and anesthetized. A dose of 100 pM TcdB/FBD (100 μL) was administered intrarectally at a depth of approximately 4 cm. To prevent toxin leakage, a rubber stick with a diameter of 2–3 mm was used to seal the anus and removed after 6 h. After 48 h, the mice were euthanized and their colons were excised for histological examination.

### Hematoxylin-eosin (H&E) staining and histopathological analysis

Colons from the mice subjected to the intrarectal instillation assay were excised, fixed in 4% paraformaldehyde, and embedded in paraffin. Tissue blocks were sectioned into slices 4 μm in thickness and stained with hematoxylin and eosin (H&E). The stained sections were evaluated in a blinded fashion by two pathologists who assessed epithelial disruption, edema, and inflammatory cell infiltration on a scale of 0 to 3, representing mild-to-severe changes. The mean scores were subsequently plotted.^6,33^

### Flow cytometry (FCM) analysis

FITC-dextran was purchased from Sigma-Aldrich (Cat# FD4-100 MG). To determine cytokine production by macrophages in the gut tissue, lamina propria cells were isolated as previously described.^52^ Colons were harvested from the mice, discarded with forceps, and washed in PBS. The gut tissue was then cut into 5–10 mm long pieces and digested with collagenase D (0.5 mg/mL; Sigma), DTT (1 mm; Sigma), and EDTA (5 mm; Sigma) in RPMI1640 complete medium for approximately 30 min at 37°C under gentle agitation. For cytokine analysis, cell suspensions were incubated 4 h in PMA/Ionomycin (Sigma-Aldrich) and Brefeldin A (Thermo Fisher) at 37°C. Intracellular staining was performed using a fixation/permeabilisation kit (BioLegends). Antibodies used for flow cytometry were obtained from Thermo Fisher or BioLegend. Cell suspensions were stained with FITC-anti-CD45 (Clone 30-F11; Cat# 103107), PerCP/Cyanine5.5-anti-CD11b (Clone M1/70; Cat# 101228), PE/Cyanine7-anti-F4/80 (Clone BM8; Cat# 123114), PE-anti-IL-1β (Clone NJTEN3; Cat# 12-7114-80), APC-anti IL-6 (Clone MP5-20F3; Cat# 504508), and Pacific Blue-anti-CD44 (Clone IM7; Cat# 103019). Macrophage cell populations were defined as CD45^+^CD11b^+^F4/80^+^. Flow cytometry was performed using a full-spectrum flow cytometer (SA3800, Sony Biotechnology, San Jose, CA, USA).

### Ethics statement

All animal procedures were approved by the Institutional Animal Care and Use Committee of Hubei University of Medicine (IACUC2021–087). To minimize distress and pain, mice were monitored hourly. Animals with signs of pain or distress, such as labored breathing, inability to move after gentle stimulation, or disorientation, were euthanized immediately. This method was approved by the IACUC and monitored by a qualified veterinarian. All applicable institutional guidelines for the care and use of the animals were followed.

### Statistics analysis

The data were analyzed with GraphPad 8.0 software. Where applicable, all quantitative data were expressed as the mean ± standard error of the mean (SEM). Two-group comparisons were statistically evaluated using an independent Student’s *t*-test, and three or more group comparisons were assessed using one-way ANOVA for statistical significance. Differences were considered statistically significant for *p* values < 0.05.

## Supplementary Material

Supplemental Material

## Data Availability

The authors confirm that the data supporting the findings of this study are available within the article [and/or] its supplementary materials.
