# Correction

**DOI:** 10.1080/19490976.2025.2532985

**Published:** 2025-07-11

**Article title**: CD44 is a macrophage receptor for TcdB from *Clostridioides difficile* that *via* its lysine-158 succinylation contributes to inflammation

**Authors**: Chen, Z., Zhang, W., Wang, D., Luo, R., Yao, Y., Tao, X., … Sun, X

**Journal**: *Gut Microbes*

**DOI**: https://doi.org/10.1080/19490976.2025.2506192

In the published version of the article, the HE staining images for the PBS control group in Figure 4f were inadvertently duplicated as the FBD-CD44-KO group in Figure 4a. These corrections have been made in the original article, and the updated version have been published. Please find below the updated Figure 4.

**Figure 4.**
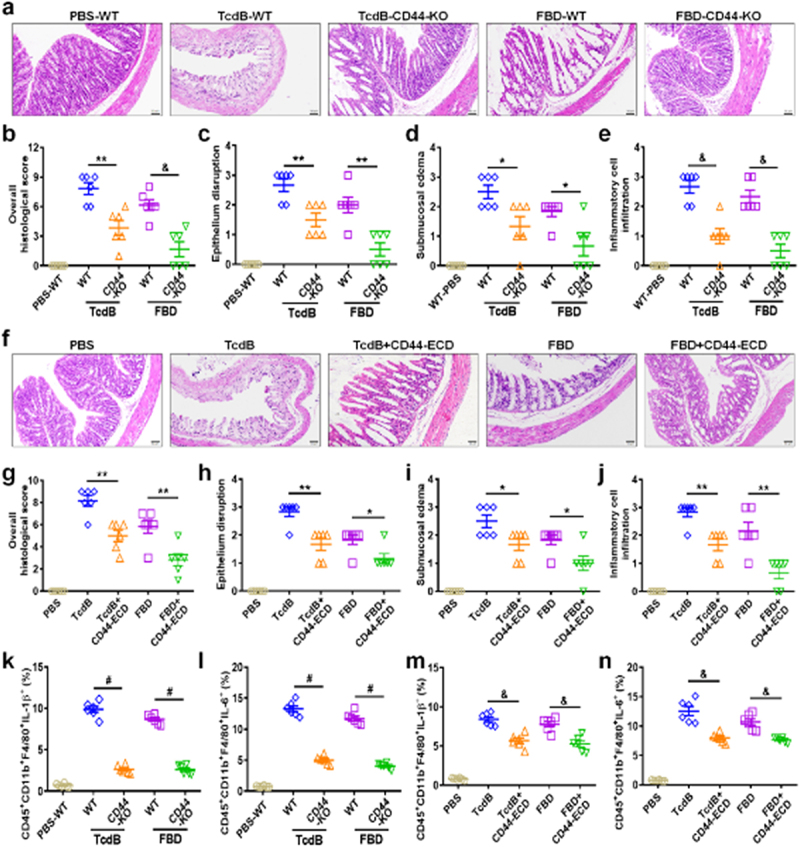
CD44 is an inflammatory-related receptor for TcdB/FBD in vivo. (a – j) mouse colonic tissues harvested after intrarectal instillation assays were assessed for pathology through H&E staining. (a) Mice colons were exposed to high concentrations of TcdB/ FBD (100 pM) after being stimulated as indicated for 48 h, and representative H&E images are shown. Scale bar represents 50 μm. (b) Overall histology scores are graphed. (c – e) histopathological scores (n = 6 mice) for (a) were assessed based on indicated pathological features for epithelium disruption (c), submucosal oedema (d), and inflammatory cell infiltration (e). (f) Mice were exposed to high concentration of TcdB/FBD (100 pM) and supersaturated CD44-ECD (1 μM), after being stimulated as indicated 48 h, and representative H&E images are shown. The scale bar represents 50 μm. (g) Overall histology scores are graphed. (h – j) histopathological scores for (f) were assessed based on indicated pathological features for epithelium disruption (h), submucosal oedema (i), and inflammatory cell infiltration (j). (k – n) FCM statistical analysis of the TcdB- or FBD-induced IL-1β (k, m) or IL-6 (l, n) production by intestinal macrophages in mice. All data in parts (b – e) and (g – n) are shown as the mean ± SEM (n = 6). (*p < 0.05, **p < 0.01, &p < 0.001, #p < 0.0001).

