# Supplementary material for: CD44 is a macrophage receptor for TcdB from Clostridioides difficile that via its lysine-158 succinylation contributes to inflammation
Source: Gut Microbes. 2025 May 18;17(1):2506192. doi: 10.1080/19490976.2025.2506192 (PMC12091907; doi:10.1080/19490976.2025.2506192)
Supplement: Supplemental Material [file KGMI_A_2506192_SM0758.zip › Table S3.docx]

**Table S3. Sequence of siRNA.**

| **Name** | **Sequence (5’-3’)** | |
| --- | --- | --- |
|  | **Sense** | **Antisense** |
| **FZD1-Homo-898** | CCGCUGAGCUCAAGUUCUUTT | AAGAACUUGAGCUCAGCGGTT |
| **FZD1-Homo-1259** | CCUCCUACCUCAACUACCATT | UGGUAGUUGAGGUAGGAGGTT |
| **FZD1-Homo-2213** | CGGUCUUCAUGAUUAAGUATT | UACUUAAUCAUGAAGACCGTT |
| **FZD2-Homo-866** | CAUCCUAUCUCAGCUACAATT | UUGUAGCUGAGAUAGGAUGTT |
| **FZD2-Homo-931** | CCCGAUGGUUCCAUGUUCUTT | AGAACAUGGAACCAUCGGGTT |
| **FZD2-Homo-1037** | CGUACUUGGUAGACAUGCATT | UGCAUGUCUACCAAGUACGTT |
| **FZD7-Homo-777** | CCAACGGCCUGAUGUACUUTT | AAGUACAUCAGGCCGUUGGTT |
| **FZD7-Homo-1068** | GCACCAUCCUCUUCAUGGUTT | ACCAUGAAGAGGAUGGUGCTT |
| **FZD7-Homo-1230** | CCGUCAAGACCAUCACUAUTT | AUAGUGAUGGUCUUGACGGTT |
| **CSPG4-Homo-4312** | GAAGGAGGACGGACCUCAAGC | UUGAGGUCCGUCCUCCUUCUG |
| **CSPG4-Homo-7993** | GUGUGGUGUUUGUGUCUUAAC | UAAGACACAAACACCACACAG |
| **CSPG4-Homo-8208** | CCUUCAGUUCAGUAGAGAAAG | UUCUCUACUGAACUGAAGGGG |
| **PVRL3-Homo-1698** | GGAUUAUUAUGAAGAUCUAAA | UAGAUCUUCAUAAUAAUCCAU |
| **PVRL3-Homo-2093** | GAAGAAAUGUCAACAUUAAAU | UUAAUGUUGACAUUUCUUCUU |
| **PVRL3-Homo-3518** | GGUUAAGGAUACAGAUAAAUA | UUUAUCUGUAUCCUUAACCUG |
| **FZD1-Mouse-1334** | CAACUCCCUCCUUGCUACCTT | GGUAGCAAGGAGGGAGUUGTT |
| **FZD1-Mouse-1469** | CCUCCUACCUCAACUACCATT | UGGUAGUUGAGGUAGGAGGTT |
| **FZD1-Mouse-1809** | CACUAAGAAAGAAGGCUGCTT | GCAGCCUUCUUUCUUAGUGTT |
| **FZD2-Mouse-1007** | GACGGCUCUAUGUUCUUCUTT | AGAAGAACAUAGAGCCGUCTT |
| **FZD2-Mouse-1242** | GCUUCUCAGAGGACGGUUATT | UAACCGUCCUCUGAGAAGCTT |
| **FZD2-Mouse-1870** | CACAGUCUACAUGAUCAAATT | UUUGAUCAUGUAGACUGUGTT |
| **FZD7-Mouse-912** | GCCAUAUCACGGCGAGAAATT | UUUCUCGCCGUGAUAUGGCTT |
| **FZD7-Mouse-1061** | UGCACCAGUUCUACCCUCUTT | AGAGGGUAGAACUGGUGCATT |
| **FZD7-Mouse-1096** | UCUCCUGAGCUACGCUUCUTT | AGAAGCGUAGCUCAGGAGATT |
| **CSPG4-Mouse-975** | GGAAAUCUCUGUAGACCAAUA | UUGGUCUACAGAGAUUUCCAG |
| **CSPG4-Mouse-4427** | CCUUUGUUCUGCUAGCUAAUG | UUAGCUAGCAGAACAAAGGCG |
| **CSPG4-Mouse-6162** | GGAUCACUUCAAAGUUGUAGC | UACAACUUUGAAGUGAUCCUG |
| **PVRL3-Mouse-1047** | GGUGUUAACCUCAAGUGUAAU | UACACUUGAGGUUAACACCUU |
| **PVRL3-Mouse-1715** | GGAUUACUAUGAAGAUCUAAA | UAGAUCUUCAUAGUAAUCCAU |
| **PVRL3-Mouse-1853** | GCGACUUCACUAUGUACAAGG | UUGUACAUAGUGAAGUCGCGU |
| **Negative Control** | UUCUCCGACAGUGUCACGUTT | ACGUGACACUGUCGGAGAATT |
