# Supplementary material for: CD44 is a macrophage receptor for TcdB from Clostridioides difficile that via its lysine-158 succinylation contributes to inflammation
Source: Gut Microbes. 2025 May 18;17(1):2506192. doi: 10.1080/19490976.2025.2506192 (PMC12091907; doi:10.1080/19490976.2025.2506192)
Supplement: Supplemental Material [file KGMI_A_2506192_SM0758.zip › Table S4.docx]

**Table S4. Sequence of oligonucleotides for qRT-PCR.**

| **Name** | **Sequence (5’-3’)** | |
| --- | --- | --- |
|  | **Forward** | **Reverse** |
| **FZD1 (Homo)** | CCAAGAGAGGAGCCGAGA | CGGCACAAAGTTCCCAG |
| **FZD2 (Homo)** | GTGCCATCCTATCTCAGCTACA | CTGCATGTCTACCAAGTACGTG |
| **FZD7 (Homo)** | CTGTCGGGCTGCTACTTCAT | GCCAGGATAGTGATGGTCTTG |
| **CD44 (Homo)** | AGAAGGTGTGGGCAGAAGAA | AAATGCACCATTTCCTGAGAC |
| **CSPG4 (Homo)** | AGCTAGCCAGGACTGATGGA | CAGCCTAACCTGCTCCAAAG |
| **PVRL3 (Homo)** | GCAGTTCACCATCCCCAATATG | TCCAAGCGGGAATGTAACAGC |
| **FZD1 (Mouse)** | TTCCTGCTGGCCGGTTTCGTGTCA | CTGGGCTCATGGGCGGGTGTGG |
| **FZD2 (Mouse)** | CCGACGGCTCTATGTTCTTC | TAGCAGCCGGACAGAAAGAT |
| **FZD7 (Mouse)** | GCCCCGACTTTACAGTCTTC | ATACCGCAGTTTCCCCCTTG |
| **CD44 (Mouse)** | TCACAAGGCAAGCACTTCAC | ATTTGGTCCATGGTGGGTAA |
| **CSPG4 (Mouse)** | AAGGAAGTGCAGAGGAGGTC | TGAGGACAGTAGGAGACCGA |
| **PVRL3 (Mouse)** | GAAGGCGAATTACTTGTGTTGTAA | TCCATCATATCCTGTTACTAAACTT |
| **GAPDH** | CCTTCCGTGTTCCTACCC | GCCTGCTTCACCACCTTC |
