# Supplementary material for: CD44 is a macrophage receptor for TcdB from Clostridioides difficile that via its lysine-158 succinylation contributes to inflammation
Source: Gut Microbes. 2025 May 18;17(1):2506192. doi: 10.1080/19490976.2025.2506192 (PMC12091907; doi:10.1080/19490976.2025.2506192)
Supplement: Supplemental Material [file KGMI_A_2506192_SM0758.zip › Supplemental material 1_Figure S1_S7.doc]

**Supplemental material 1**

**Contents**

**Figure S1. E**ffect of FBD-mediated macrophage secretion of inflammatory cytokines is comparable to that of TcdB, but not CROPs.

**Figure S2.** TcdB/FBD stimulated the production of IL-1β and IL-6 by macrophages independent of FZD1/2/7, CSPG4, and PVRL3.

**Figure S3.** TcdB/FBD induced IL-1β and IL-6 production in macrophages through a CD44-dependent mechanism.

**Figure S4.** Representative FACS plots of the TcdB- or FBD-induced IL-1β or IL-6 production in murine intestinal macrophages by flow cytometry analysis.

**Figure S5.** Flow cytometry was performed to assess CD44^+^ macrophage responses to TcdB/FBD stimulation in the murine intestine.

**Figure S6.** Representative flow cytometry histograms illustrating TcdB/FBD-mediated modulation of macrophage phagocytosis through CD44.

**Figure S7.** CD44 subcellular localization analysis in lipid-raft domains under different stimulation conditions.

**Figure S1. Effect of FBD-mediated macrophage secretion of inflammatory cytokines is comparable to that of TcdB, but not CROPs. (a)** Extracted CROPs were analyzed by SDS−PAGE. Lane 1, marker; Lane 2, CROPs with un-cleaved 6×His/SUMO tag; Lane 3, CROPs without 6×His/SUMO tag. **(b)** THP-1-M*φ* were treated with different proteins, such as TcdB, FBD, and CROPs, for 24 h and then the production of IL-6 were measured by an ELISA. **(c)** THP-1-M*φ* were treated with 100 pM of TcdB, FBD, or CROPs, and the levels of IL-6 was assessed at different time points. **(d)** THP-1-M*φ* were treated with TcdB/FBD (100 pM), after being pre-stimulated or stimulated directly as indicated for 24 h, and the levels of IL-6 were measured. **(e)** Representative microscopy images over time of Caco-2 cells co-cultured with THP-1-M*φ*, which were pre-stimulated or stimulated directly with TcdB/FBD, were analyzed by an HCI system. All data in parts (b–d) are shown as the mean ± SEM (n = 3). (ns: *p* > 0.05, *: *p* < 0.05, **: *p* < 0.01, &: *p* < 0.001, #: *p* < 0.0001; b: *vs* 0 pM; c: *vs* 0 h).

**Figure S2.** **TcdB/FBD stimulated the production of IL-1β and IL-6 by macrophages independent of FZD1/2/7****, CSPG4, and PVRL3.** siRNA screening of **(a)** human FZD1, **(b)** FZD2, and **(c)** FZD7, **(d)** CSPG4, and **(e)** PVRL3. siRNA screening of mouse **(f)** FZD1, **(g)** FZD2, and **(h)** FZD7, **(i)** CSPG4, and **(j)** PVRL3. **(k–m)** The macrophages were treated with TcdB or FBD (100 pM) for 24 h after knockdown of FZD1/2/7, **(k)** production of IL-6 by the THP-1-M*φ* was determined, and the production of **(l)** IL-1β and **(m)** IL-6 by the BMDMs was determined by ELISA. **(n–q)** The macrophages were treated with TcdB or FBD (100 pM) for 24 h after knockdown of CSPG4 or PVRL3, the production of **(n)** IL-1β and **(o)** IL-6 by the THP-1-M*φ* was determined, and the production of **(p)** IL-1β and **(q)** IL-6 by the BMDMs was determined by ELISA. NC: Negative Control. The red boxes in (a–j) represent siRNAs that effectively knocked down human or mouse FZD1/2/7, CSPG4, and PVRL3. All data are shown as the mean ± SEM (n = 3). (ns: *p* > 0.05, *: *p* *<* 0.05, **: *p* *<* 0.01, &: *p* *<* 0.001, #: *p* *<* 0.0001; a–j: *vs* NC).

**Figure S3. TcdB/FBD induced IL-1β and IL-6 production in macrophages through a CD44-dependent mechanism. (a–c)** Macrophages were treated with TcdB/FBD (100 pM) and CD44-ECD (1 μM) for 24 h. **(a)** The production of IL-6 by THP-1-M*φ* was determined, and the production of **(b)** IL-1β and **(c)** IL-6 by BMDMs was determined by ELISA. **(d–f)** WT and CD44-knockout macrophages were treated with TcdB or FBD (100 pM) for 24 h. **(d)** The production of IL-6 by THP-1-M*φ* was determined, and the production of **(e)** IL-1β and **(f)** IL-6 by BMDMs was determined by ELISA. **(g)** Wild-type or CD44-mutated THP-1-M*φ* were treated with TcdB or FBD (100 pM) for 24 h, and the production of IL-6 was determined by ELISA. All data are shown as the mean ± SEM (n = 3). (ns: *p* > 0.05, *: *p* *<* 0.05, **: *p* *<* 0.01, &: *p* *<* 0.001, #: *p* *<* 0.0001).

**Figure S4. Representative FACS plots of the TcdB- or FBD-induced IL-1β or IL-6 production in murine intestinal macrophages by flow cytometry analysis. (a)** Intestinal macrophages were isolated from mice and analyzed by flow cytometry. Macrophages cell populations were defined as CD45^+^CD11b^+^F4/80^+^. **(b–c)** Representative FACS plots of the TcdB- or FBD-induced IL-1β **(b)** or IL-6 **(c)** production in murine intestinal macrophages in wild-type (WT) and CD44-knockout (KO) mice. **(d–e)** Representative FACS plots of the TcdB- or FBD-induced IL-1β **(d)** or IL-6 **(e)** production with or without CD44 extracellular domain (CD44-ECD) by murine intestinal macrophages.

**Figure S5. Flow cytometry was performed to assess CD44^+^ macrophage responses to TcdB/FBD stimulation in the murine intestine. (a)** Representative FACS plots showing CD44 expression in IL-1β^+^ or IL-6^+^ macrophages after TcdB/FBD treatment. **(b)** Quantification of CD44 mean fluorescence intensity (MFI) in intestinal macrophages. **(c)** Changes in total macrophage population following TcdB/FBD exposure. All data in parts (b, c) are shown as the mean ± SEM (n = 6). (&: *p* *<* 0.001, #: *p* *<* 0.0001).

**Figure S6. Representative flow cytometry histograms illustrating TcdB/FBD-mediated modulation of macrophage phagocytosis through CD44. (a–b)** FITC-dextran uptake assay in THP-1-M*φ* **(a)** and BMDMs **(b)** from WT and CD44-knockout macrophage**s**. Cells were treated with 100 pM TcdB or FBD for 24 hours, followed by exposure to FITC-dextran (250 μg/ml) for 1 hour. **(c–d)** FITC-dextran uptake assay in THP-1-M*φ* **(c)** and BMDMs **(d)** co-treated with 100 pM TcdB/FBD and 1 μM CD44-ECD for 24 hours, then incubated with FITC-dextran (250 μg/ml) for 1 hour. Fluorescence intensity was quantified by flow cytometry.

**Figure S7. CD44 subcellular localization analysis in lipid-raft domains under different stimulation conditions. (a)** Western blot analysis of CD44 expression in THP-1-M*φ* stimulated with or without TcdB/FBD (left). Quantitative analysis of CD44 distribution ratios (lipid-raft *vs*. non-lipid-raft fractions) (right). **(b–c)** CD44 expression in CD44-mutated THP-1-M*φ* treated with TcdB (b) or FBD (c) (left), with corresponding lipid-raft/non-lipid-raft CD44 ratios (right). **(d)** Western blot detection of CD44 variants (WT, K158R [de-succinylation mimic], K158E [succinylation mimic]) in THP-1-M*φ* CD44-KO cells (left). Quantitative comparison of their lipid-raft partitioning (right). Flotillin-1 served as lipid-raft marker (positive control). All data are shown as the mean ± SEM (n = 3).（ns：*p*>0.05）.
